# Distribution Characteristics and Source of Dechloranes in Soil and Lichen of the Fildes Peninsula (Antarctica)

**DOI:** 10.3390/ijerph15102312

**Published:** 2018-10-21

**Authors:** Hui Gao, Guangshui Na, Yao Yao, Ruijing Li, Yuhang Gao, Zhifeng Zhang, Ziwei Yao

**Affiliations:** 1 National Marine Environmental Monitoring Center, Dalian, Liaoning 116023, China; hgao@nmemc.org.cn (H.G.); oceanyaoyao@163.com (Y.Y.); liruijing158@163.com (R.L.); yhgao93@163.com (Y.G.); zfzhang@nmemc.org.cn (Z.Z.); zwyao@nmemc.org.cn (Z.Y.); 2 State Key Laboratory of Marine Environmental Science, College of Ocean and Earth Sciences, Xiamen University, Xiamen 361102, China; 3 College of Marine Sciences, Shanghai Ocean University, Shanghai 201306, China

**Keywords:** dechloranes, stereo selection, soil, lichen, Fildes Peninsula

## Abstract

Dechloranes (Decs) have been widely found in the environment, even in the Tibetan Plateau and remote polar regions. However, the understanding of their regional distribution characteristics in polar regions is limited. To study the long-range atmospheric transport and fates of these emerging contaminants, Decs were analyzed in soil and lichen from the Fildes Peninsula in Antarctica. The concentrations of five Decs in soil and lichen ranged from 141.46 to 838.47 pg/g dw and 237.04 to 3599.18 pg/g dw, respectively. The mean fractions of *anti*-Dechlorane Plus (DP) (*f*_anti_) values estimated in the current soils (0.37) and lichen (0.24) were lower than those of commercial products (*f*_anti_ = 0.64–0.80), which confirms that long-range atmospheric transport is a main source of DP, and the DP burdens could be driven by the accumulation of *syn*-DP. The average ΣDP concentration in soil in the coastal area was higher than that in the inland area and Ardley Island, while in lichen, the average DP concentration at the Ardley Island site was approximately three-fold higher than that in the coastal area and inland areas. This indicates that the distribution of DP was influenced by anthropogenic interference and animal activities in the Fildes Peninsula. The spatial variation of *f*_anti_ of the three regions was clearer in soil than that in lichen. The *f*_anti_ values were negatively correlated with DP concentrations in soil, suggesting that DP concentration levels play an important role in determining the isomeric composition of DP in the soil.

## 1. Introduction

Dechlorane (Dec) 602, Dec 603, Dec 604, and Dechlorane Plus (DP) are highly chlorinated flame retardants that have been used to replace Mirex, which is an organochloride that was banned because of its toxicity and negative impact upon the environment [1]. Decs are extensively used in consumer products, including electronic equipment, textiles, furniture, and automobiles, to increase their resistance to fire [2]. DP exists as two stereoisomers (*syn*- and *anti*-DP), which are mixed in a ratio of 1:3 in commercial products [3]. DP possesses characteristics similar to those of persistent organic pollutants (POPs), and it has become a ubiquitous pollutant in the environment. Since the first report of DP occurrence in one of the North American Great Lakes in 2006, research has been carried out to investigate the occurrence of DP in various environmental matrices, including air [4,5], tree bark [6,7], water [4,8], sediment [9,10,11], soils [12,13], dust [3], sewage sludge [14], aquatic organisms [5,15], birds [1,16], human hair, and serum [17,18] in all of the major continents, except Africa [19]. A few studies have published environmental data regarding the presence of Dec 602, Dec 603, and Dec 604 in sediments and aquatic biota in the Great Lakes [10,20], and the shore area of northern China [21]. However, the data on the occurrence and distribution of Decs in polar regions is still limited.

The fastest and most direct way for the pollutants to reach polar regions is though atmospheric transport [4], which can take place over days or weeks, and it has been recognized as the main source of organic pollutants in polar regions. Polycyclic aromatic hydrocarbons (PAHs), polychlorinated biphenyls (PCBs), polybrominated diphenyl ethers (PBDEs), and organochlorine pesticides (OCPs) have all been reported in polar regions in different environmental media. Evidence of Decs in polar regions suggests that they are recalcitrant to photolysis and biodegradation [22,23], which may influence the regional characteristics of Decs. Based on *f*_anti_, Möller found that DP can undergo long-range atmospheric transport to remote Arctic and Antarctic regions [4,23]. Therefore, the *f*_anti_ in environmental matrices was used to indicate the source of DP in certain areas.

As a major sink of pollutants, the polar regions have been recognized as important areas that indicate the environmental behavior of POPs. Since there are no obvious sources of pollution in the polar regions, they are becoming a sink of POPs and may be used as an indicator of global pollution change [24]. The “exogenous” feature of polar pollutants provides a good platform for studying the environmental behavior of contaminants. The Fildes Peninsula (58°57′51.9″ W, 62°12′59.7″ S) is located on the southern end of King George Island in the South Shetland Islands of Antarctica, which has a Russian research station, the Great Wall Station, and an airport, and therefore human activity is relatively frequent. Penguins gather at the nearby Ardley Island, and thus, animal activity is also relatively frequent in this area. Surface soil and lichen in the Fildes Peninsula were analyzed in this study to provide the concentration level, distribution characteristics, and sources of Decs. The results of this study will help in investigating the sources and the fate of Decs in the Fildes Peninsula.

## 2. Experimental Section

### 2.1. Sampling

**Figure 1:**
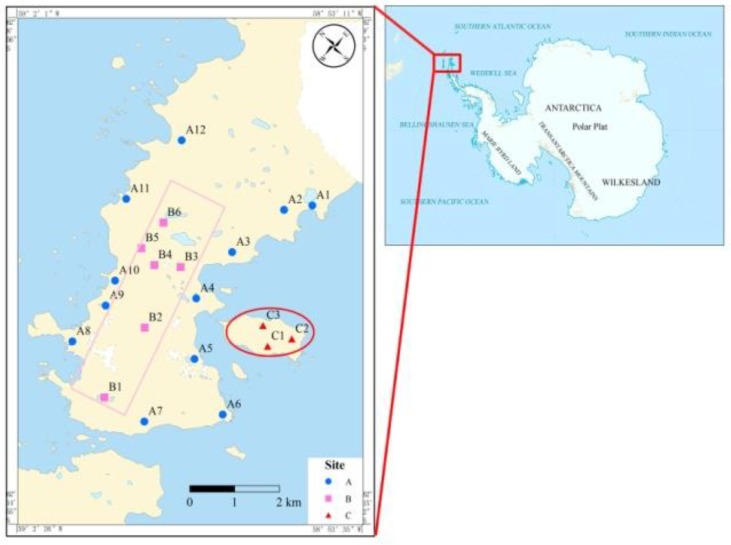
Map of the sampling sites.

A total of 20 soil and 18 lichen samples were taken from January to March, 2014 in the Fildes Peninsula, Antarctica. In order to better explore the regional distribution characteristics of Decs, the Fildes Peninsula was divided into the coastal area, inland area, and Ardley Island, to consider different human and animal activity levels. A map of the sampling areas is presented in Figure 1. Soil and lichen samples were freeze-dried and then sieved (80-mesh) prior to analysis.

### 2.2. Chemical Analysis

Before extraction, an internal standard of polychlorinated biphenyl 209 (99%) was added to all of the samples as surrogate compounds Approximately 5 g of soil or lichen samples mixed with the surrogate standard was extracted via accelerated solvent extraction with 50 mL hexane/dichloromethane (DCM) (1:1, *v*/*v*). Activated copper powder was added to the extracts of soil to remove elemental sulfur. These samples were Soxhlet-extracted for 24 h using hexane/DCM (1:1, *v*/*v*). The raw extracts were dried to 5 mL with a rotary evaporator and were transferred to a multi-layer column filled from the bottom with 2 g activated silica gel, 4 g neutral alumina, and 1 cm anhydrous Na_2_SO_4_ (pre-soaked in hexane). The extracts were then eluted with 70 mL hexane/DCM (1:1, *v*/*v*) and were further evaporated under a gentle N_2_ stream. The samples were solvent-exchanged to hexane (50.0 μL).

The Dec samples were analyzed on an Agilent 6890N gas chromatograph (GC, Santa Clara, CA, USA) coupled with a 5973I mass spectrometer (MS, Agilent Technologies, Inc., Santa Clara, CA, USA) using negative chemical ionization mode with methane as the ionization gas. The GC was fitted with a DB-5HT capillary column (0.25 mm i.d. × 15 m × 0.10 μm film thickness, J&W Scientific, Inc., Folsom, CA, USA). The gas chromatography oven was programmed as follows: initial 80 °C for 2 min, 20 °C/min until 180 °C, 5 °C/min until 250 °C and held for 2 min, 30 °C/min until 310 °C and held for a final 5 min. The MS transfer line was held at 280 °C. The temperature of the ion source and the quadrupole was 150 °C.The extract (1.0 µL) was injected in pressure-pulsed splitless mode. Helium was used as the carrier gas at a flow rate of 1.0 mL/min. The instrument was operated in selected ion monitoring mode (*m*/*z* 606.0, 608.2, and 610.0 for Dec 602; *m*/*z* 628.0, 630.0, and 634.0 for Dec 603; *m*/*z* 569.0, 608.0, and 612.0 for Dec 604; and *m*/*z* 646.0, 645.0, and 649.0 for *syn*-DP and *anti*-DP).

### 2.3. Quality Assurance and Quality Control

Three reagent blanks and matrix spikes were subject to the same procedures along with the samples. The average recoveries for Decs were 98% to 110%, respectively. The mean recoveries of the surrogate standard (CB 209) was 80% to 113%. The method detection limits (MDLs) were calculated as the signal that was three times that of the noise level. The MDLs of Decs ranged from 0.19 to 24.04 pg/g in soil samples and from 0.05 to 0.90 pg/g in lichen samples.

### 2.4. Statistics

All of the data were analyzed using SPSS 19.0 (International Business Machines, Armonk, NY, USA) software. Linear regression analysis was used to determine the relationship between the *f*_anti_ values and DP levels. For concentrations below the MDL, the result was treated as half the level of the MDL.

## 3. Results and Discussion

### 3.1. Levels and Source of Decs in Soil and Lichen

The DP concentrations in soil samples at 20 sampling sites in the Fildes Peninsula are presented in Table 1. DP was detected in all of the soil samples, with a mean concentration of 212.25 pg/g dry weight (dw) and a range from 57.86 to 336.42 pg/g dw. These data illustrate that there were some residues of DP in the soil of the Fildes Peninsula. The data for DP levels in soils are very limited, and little comparison can therefore be made with the results of available studies. However, detected concentrations of DP in the surface soil samples collected from an industrial region (0.0336–4.65 ng/g) and e-waste recycling (nd–47.4 ng/g) in South China [25] were higher than the levels detected in the current study. The DP concentrations in the current study were between one and several orders of magnitude lower than those in the aforementioned studies. The sources of DP mainly include manufacturing plants, as well as the emissions from DP-containing e-waste recycling activities and the use of DP-containing products [26], and these may account for the low level of DP in the Fildes Peninsula. Moreover, DP is recalcitrant to photolysis and biodegradation, and it has long-range transport potential in addition to biomagnification and bioaccumulation potential [4,22], indicating that long range transport is likely the main source of DP in the Antarctica, as previously reported [23].

Lichen is an easy and inexpensive monitor for semi-volatile organic compounds [27,28,29]. It is currently used as a typical, natural passive air sampler to indicate atmospheric pollution [30,31]. At present, minimal or no information on Decs levels in plants has been reported in polar regions. Decs were detected in lichen samples from the Fildes Peninsula (Table 1). The DP concentrations ranged from 175.00 to 3048.91 pg/g dw, with a mean value of 589.31 pg/g dw. The average concentrations of DP in lichenin this study were similar to those in reeds in northeastern China (0.63 ng/g dw) [32] and in lichen in the southeast Tibetan Plateau (167 pg/g) [33]. However, the concentrations found in this study were lower than those in tree bark from the northeastern US (0.03–115 ng/g) and Korea (1.4 ng/g), which were influenced by DP manufacturing facilities [7]. However, DP concentrations in this study were significantly higher than those in moss from the remote Ny-Ålesund, Norway, above the Arctic Circle [23]. Furthermore, we found that the DP concentrations in lichen were higher than those in soil. This is probably because lichen grow slowly and are exposed to pollutants for a long time in the air and thus will adsorb more DP. However, biodegradation occurs in the soil, reducing DP concentrations.

Other DP-like compounds, including Dec 602, Dec 603, and Dec 604, were all detected in soil from the Fildes Peninsula. The concentrations and distributions of Dec 602, Dec 603, and Dec 604 are shown in Figure 1 and Table 1. Dechlorane 604 was detected in 13 of the 20 soil samples, with a mean concentration of 146.44 pg/g dw (ranging from below the detection limit (BDL) to 483.22 pg/g dw). The Dec 603 concentrations ranged from BDL to 74.25 pg/g dw with a mean value of 24.43 pg/g dw, and Dec 602 concentrations were higher than BDLin only eight soil samples, ranging from BDL to 7.04 pg/g dw with a mean value of 2.36 pg/g dw. The concentrations of Dec 602, Dec 603, and Dec 604 in soil from the Fildes Peninsula were comparable to those in soils from Ny-Ålesund [23].

The Dec 602, 603, and 604 concentrations in lichen have been previously reported at Ny-Ålesund [23] and northern China [32]. In the current study, Dechlorane 602 and Dec 603 concentrations were higher than those of BDL in only three and four lichen samples, respectively. Dec 604 was detected in all of the lichen samples. The mean concentrations of Dec 602, Dec 603, and Dec 604 in lichen were 0.78 pg/g dw, 13.81 pg/g dw, and 107.25 pg/g dw, respectively. The concentrations of Dec 603 were comparable to those reported for the North China Sea (Dec 603, detected in two out of five samples, ranging from BDL to 0.024 ng/g wet weight (ww), with a mean of 0.009 ± 0.010 ng/g ww) [32].

### 3.2. Spatial Distribution of Dechloranes in Soil and Lichen

In order to determine how Decs are distributed in soil of the Fildes Peninsula, the soil sampling sites in the Fildes Peninsula included 12 coastal sites, five inland sites, and three Ardley Island sites. The DP concentrations in the three types of sampling site are provided in Table 1. The average DP concentration at the closest sites of the coastal area was higher than that in inland areas and the Ardley Island area (Coastal > Inland > Ardley Island). This may have resulted from the different anthropogenic interference in the three areas. In coastal areas, there were scientific investigation stations, oil depots, and airports, which may bring in DP along with construction materials. Moreover, some of the soil sampling sites are located near where glaciers melt and water flows through, and thus, DP in snow and lake water will be deposited on the surface of the soil. This may have caused the high concentration of DP in the coastal area. In inland areas, the sampling sites were mostly located near valleys and peaks, with relatively lower human activities. The Ardley Island area is the main gathering place of penguins and seals. This may explain why the concentrations of DP in the inland area were higher than those of the Ardley Island area. This suggests that the distribution of DP in the Fildes Peninsula might be primarily influenced by local human activity, station base construction, and lake and meltwater flows.

The DP concentration at the A5 site in the coastal area was significantly higher than that in the Ardley Island area and inland areas. Site A5 was located at the base station, which is the most concentrated area of human activity in the polar region. This clearly indicates that human activity has a certain effect on the distribution of DP in the polar region. Consequently, the base station site may become a primary sink of pollutants. Various types of contaminations, such as hydrocarbons [34], heavy metals [35], and persistent organic pollutants [36], have been detected in soil, meltwater stream sediments, and benthic organisms in Antarctica. Relatively low concentrations were detected at Ardley Island site A10, which is distant from areas of human activity. The analysis results supports these conclusions.

The distribution of Dec 604 was similar to that of DP, indicating that its concentrations in soil from the coastal area were significantly greater than those from the inland area and Ardley Island. In contrast to DP and Dec 604, the concentration of Dec 603 in soil from the coastal area was lower than that in the inland area, and Dec 603 was detected on Ardley Island. The concentration of Dec 602 was similar in the three areas, and was lower than Dec 603, Dec 604, and DP.

The lichen sampling sites in the Fildes Peninsula of Antarctica included nine coastal sites, six inland sites, and three Ardley Island sites. As shown in Table 1, the DP concentrations at the Ardley Island sites were 4–5 times higher than those of coastal or inland area sites, indicating a higher concentration of DP in plants at the Ardley Island area. An additional study was conducted to investigate the influence of air on the distribution of dechloranes in lichen in the polar region [37]. Similar to the conclusions obtained from the study of soil, there were higher levels of local human activity, station base construction, and lake and meltwater in the coastal and inland areas compared with the Ardley Island sites. Thus, the volume of DP discharged into the coastal and inland areas could be higher than that discharged into Ardley Island. It was hypothesized that the concentrations of DP in the Ardley Island area should be lower than those. However, the results were contradictory with the above hypothesis because the mean concentration of DP was 449.32 pg/g dw and 337.47 pg/g dw in the coastal and inland areas, respectively, while the mean was 1513.03 pg/g dw at Ardley Island. This may be because DP is a semi-volatile organic compound, and penguin excrement volatilizes DP. Lichens grow slowly and are exposed to pollutants for a long time, thus adsorbing more DP, which results in a higher concentration of DP in lichen in penguin-dense Ardley Island than the other two areas.

The distribution of Dec 604 was similar to that of DP, indicating that their concentrations in lichen from Ardley Island were significantly greater than those from the coastal and inland areas. In contrast to DP and Dec 604, the concentrations of Dec 602 and 603 in lichen from the Ardley Island area were lower than those in the coastal and inland areas. The mean concentration of Dec 602 was the lowest in the coastal area sites, whereas that of Dec 604 was the highest.

### 3.3. Fractional Abundance of DP Isomers

**Figure 2:**
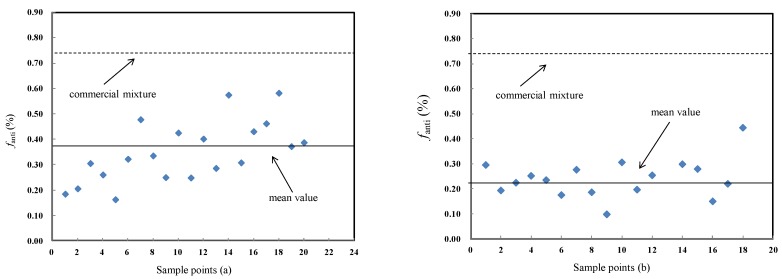
The *f*_anti_ values of (**a**) soil and lichen soil and (**b**) lichen from the Fildes Peninsula, Antarctica.

The fraction of anti-DP (*f*_anti_), defined as the amount of anti-DP divided by the sum of *syn*- and *anti*-DP concentrations, was calculated to evaluate the possible stereoisomer selective enrichment of DP isomers in soil and lichen. The *f*_anti_ values ranged from 0.16 to 0.58 and from 0.10 to 0.44 in soil and lichen, respectively, indicating large variations (Figure 2). The mean *f*_anti_ values estimated in the current soils (0.37) and lichen (0.24) are lower than those detected in the technical DP (0.64–0.80) and many other environmental samples [6,8,38,39,40]. This suggests that the DP burdens in the soil and lichen could be driven by the accumulation of *syn*-DP, indicating a stereo selection with an enrichment of the *syn*-isomer during atmospheric transport, which is consistent with the findings of Möller [4,22]. However, it is contrary to the findings in soil, plants, and fish in China, where the DP burden seemed to be substantially driven by the preferential accumulation of *anti*-DP [6,25,32], which may have occurred due to the local DP sources such as manufacturers in these regions. This could be attributed to the isomer-specific uptake of DP by the lichen and the absorption of soil from the air, or the difference in physicochemical properties of *anti*-and *syn*-DP, such as log *K_OA_* and solubilities (Sverko et al. [4,19,22]). Unfortunately, little information on isomer-specific physicochemical parameters of DP is available in the literature.

**Figure 3:**
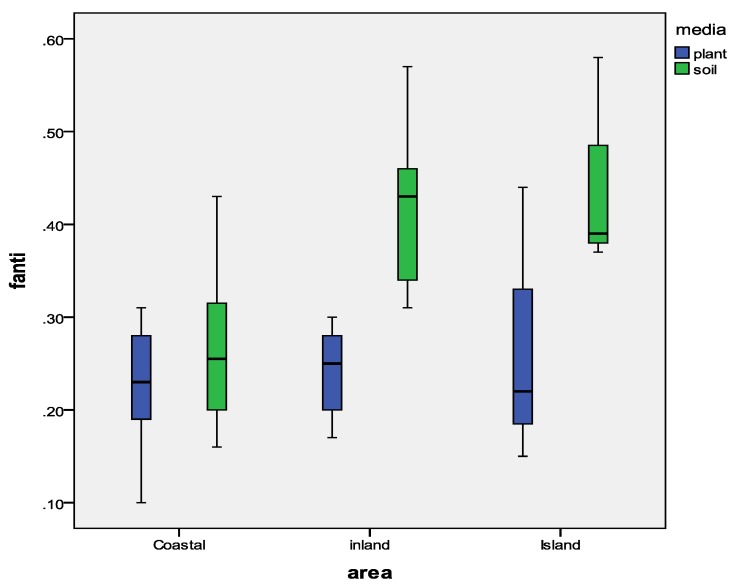
Boxplot of the *f*_anti_ values in soil and lichen from the Fildes Peninsula, Antarctica.

**Figure 4:**
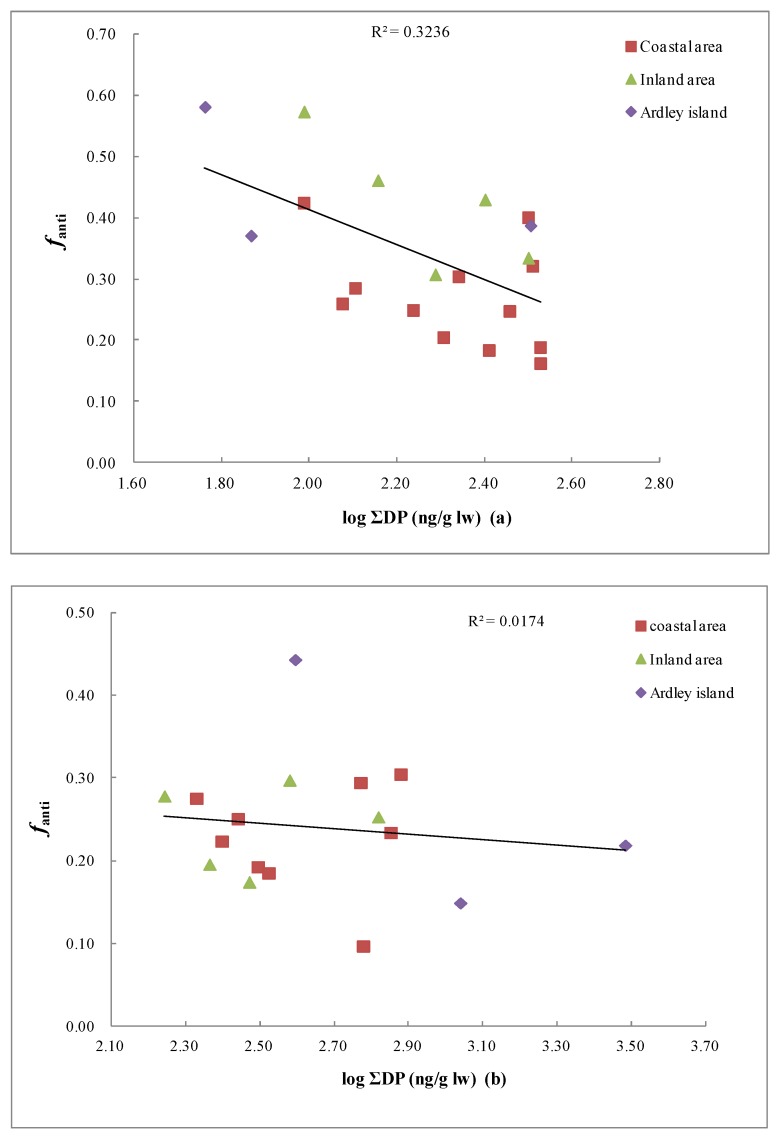
Correlation between the *f*_anti_ values and DP levels in (**a**) soil and (**b**) lichen from the Fildes Peninsula, Antarctica.

The fraction of *anti*-DP (*f*_anti_) was calculated to evaluate the possible stereoisomer selective enrichment of DP isomers in the Fildes Peninsula, Antarctica. The mean *f*_anti_ values in soil and lichen of the Fildes Peninsula of the coastal area, inland area, and Ardley Island were 0.27 and 0.24, 0.42 and 0.23, and 0.45 and 0.27, respectively (Figure 3). The *f*_anti_values of the three areas were different in soil, but the *f*_anti_ values in lichen were not significantly different. The reasons for this appearance of regional changes were not immediately apparent. In order to determine the reason for this spatial variation, we conducted a correlation analysis between the *f*_anti_ values and DP concentrations in soil. The results show a significant negative relationship between *f*_anti_ values and DP concentrations (*p* < 0.01) (Figure 4), indicating that the DP level might also be an important factor for determining the isomeric composition. This was consistent with the results for bird eggs [41], However, there was no significant correlation between the *f*_anti_ values and DP concentrations in lichen (*p* > 0.05). Lichen may be more strongly influenced by the atmosphere because it is an aerial plant. Moreover, a previous study revealed that the fate of DP was also influenced by the metabolism and assimilation efficiency of organisms [42].

## 4. Conclusions

This study showed that Decs were widely detected in soil and lichen samples, and that they were brought to the Fildes Peninsula, Antarctica, by long-range atmospheric transport. The concentration and regional characteristics of DP in soil and lichen were significantly different, indicating that regional characteristics of DP were affected by anthropogenic and animal activities. The *f*_anti_ values were negatively correlated with DP concentrations in soil, indicating that the residual content of DP in the soil was a factor influencing the *f*_anti_ value. This research investigated the distribution and transformation of DP in soil and plants to reveal the source and fate of DP in the Fildes Peninsula. In future research, we will focus on the global environmental behavior of Decs to provide evidence that can be utilized for pollutant control and management.

## Figures and Tables

**Table 1:** Concentrations (mean values) of dechloranes in soil and lichen samples from coastal, inland, and Ardley island areas in the Fildes Peninsula, Antarctica.

Sampling Area	Sample Number		Soil (pg/g dw)				Sample Number		Lichen (pg/g dw)			
			602	603	604	DP			602	603	604	DP
Coastal area	12	FD (%)	33	25	75	100	9	FD (%)	22	22	78	100
		Range	nd–7.04	nd–43.10	nd–483.22	97.18–336.42		Range	nd–1.72	nd–62.91	nd–309.70	213.29–757.12
		Mean	2.31 ± 2.01	21.01 ± 10.89	187.85 ± 147.60	232.58 ± 88.96		Mean	0.65 ± 0.44	18.09 ± 23.90	69.64 ± 93.81	449.32 ± 213.26
Inland area	5	FD (%)	40	60	40	100	6	FD (%)	17	33	83	100
		Range	nd–5.56	nd–74.25	nd–116.85	97.37–316.08		Range	nd–3.56	nd–24.74	nd–130.76	175–659.24
		Mean	2.49 ± 2.01	38.20 ± 25.02	52.89 ± 38.94	200.54 ± 86.48		Mean	0.97 ± 1.27	11.27 ± 8.28	70.16 ± 46.29	337.47 ± 171.90
Ardley island	3	FD (%)	67	0	67	100	3	FD (%)	33	0	100	100
		Range	nd–3.05	nd	nd–293.87	57.86–319.71		Range	nd–1.47	nd	142.14–543.75	393.80–3048.91
		Mean	2.33 ± 1.06	nd	136.73 ± 139.39	150.43 ± 146.82		Mean	0.79 ± 0.59	nd	294.26 ± 217.79	1513.03 ± 1375.72

Note: FD, frequency of detection; DP, Dechlorane Plus; nd, not detected.
